# Development of a novel prognostic score combining clinicopathologic variables, gene expression, and mutation profiles for lung adenocarcinoma

**DOI:** 10.1186/s12957-020-02025-0

**Published:** 2020-09-19

**Authors:** Guofeng Li, Guangsuo Wang, Yanhua Guo, Shixuan Li, Youlong Zhang, Jialu Li, Bin Peng

**Affiliations:** 1 Department of Thoracic Surgery, Shenzhen People’s Hospital, Second Clinical Medical College of Jinan University, Luohu District, Shenzhen, 518020 China; 2 Department of Thoracic Surgery, Shanghai Pulmonary Hospital, School of Medicine, Tongji University, Yangpu District, Shanghai, 200433 China; 3 Department of Biostatistics, HuaJia Biomedical Intelligence, Shenzhen Overseas Chinese High-Tech Venture Park, Nanshan District, Shenzhen, 518057 China

**Keywords:** Prognosis, Gene expression profiles, Lung adenocarcinoma, Competing risks analysis, Risk stratification, Event-free survival, Recurrence-free survival, Integrative analysis

## Abstract

**Background:**

Integrating phenotypic and genotypic information to improve prognostic prediction is under active investigation for lung adenocarcinoma (LUAD). In this study, we developed a new prognostic model for event-free survival (EFS) and recurrence-free survival (RFS) based on the combination of clinicopathologic variables, gene expression, and mutation data.

**Methods:**

We enrolled a total of 408 patients from the Cancer Genome Atlas Lung Adenocarcinoma (TCGA-LUAD) project for the study. We pre-selected gene expression or mutation features and constructed 14 different input feature sets for predictive model development. We assessed model performance with multiple evaluation metrics including the distribution of C-index on testing dataset, risk score significance, and time-dependent AUC under competing risks scenario. We stratified patients into higher- and lower-risk subgroups by the final risk score and further investigated underlying immune phenotyping variations associated with the differential risk.

**Results:**

The model integrating all three types of data achieved the best prediction performance. The resultant risk score provided a higher-resolution risk stratification than other models within pathologically defined subgroups. The score could account for extra EFS-related variations that were not captured by clinicopathologic scores. Being validated for RFS prediction under a competing risks modeling framework, the score achieved a significantly higher time-dependent AUC as compared to that of the conventional clinicopathologic variables-based model (0.772 vs. 0.646, *p* value < 0.001). The higher-risk patients were characterized with transcriptional aberrations of multiple immune-related genes, and a significant depletion of mast cells and natural killer cells.

**Conclusions:**

We developed a novel prognostic risk score with improved prediction accuracy, using clinicopathologic variables, gene expression and mutation profiles as input, for LUAD. Such score was a significant predictor of both EFS and RFS.

**Trial registration:**

This study was based on public open data from TCGA and hence the study objects were retrospectively registered.

## Background

Lung cancer is the most frequently diagnosed cancer and the leading cause of cancer death, with a total of 2,093,876 new cases (11.6% of all cancers) and 1,761,007 deaths (18.4% of all cancers) reported worldwide in 2018 [1]. LUAD is a major type of primary lung cancer which accounts for about 35% of all cases [2]. Improving survival of lung cancer is of high importance since the 5-year survival rate remains < 15% and 10-year survival rate < 7% [3]. Currently, clinicopathologic factors including American Joint Committee on Cancer (AJCC) tumor stage, tobacco smoking history and radiation therapy are used for prognostic analysis. However, whether the prediction performance of these clinicopathologic factors can be improved with phenotypic and genotypic profiles at gene level is still under investigation.

The high-throughput sequencing technology has made it possible a comprehensive interrogation of whole transcriptome and genome of tumor tissues at an increasingly reasonable cost [4, 5]. Previous studies focused on finding prognostic signatures based on gene expressio n[6–9] or mutation [10–12] for LUAD patients. For example, Li et al. [7] reported gene expression-based models with an average C-index of 0.604 on testing datasets from TCGA-LUAD in predicting overall survival (OS). Other studies using multiple types of input data made statistical inference on the significance of potential individual prognostic factors [13–16]. Two of these studies [15, 16] had shown clear benefit of combining genetic mutations and expression profiles in predicting OS and RFS at cross-validation level. In particular, they inferred that the genotype and expression data made around 5% and 50% relative contributions to explained variance of survival outcomes [16].

In this study, we aimed to improve prognostic prediction for LUAD by extensively integrating clinicopathologic variables, gene expression and mutation profiles as the input. We focused on the analysis of recurrence and death events as there exists minimal ambiguity in the database about the derivation of these outcomes [17]. We believe that our work will be informative for those who want to improve the precision treatment of LUAD.

## Methods

### Data

The study enrolled from the Cancer Genome Atlas lung adenocarcinoma (TCGA-LUAD) project [18] a total of 408 patients with relatively complete information in high-throughput DNA and RNA sequencing data, major clinicopathologic variables (at most 10 missing values was allowed), and follow-up data for recurrence or death events. The RNA expression was measured on a total of 60,483 genes and the somatic mutation was detected among 16,980 genes for each patient. The study cohort included a total of eight clinicopathologic variables: age of initial diagnosis, gender, tobacco smoking status, AJCC tumor stage, adjuvant radiation treatment, adjuvant pharmaceutical treatment, history of other malignancies, and the anatomic position of tumor (Table S1). For missing value imputation, we used the mean estimate for continuous variables and multinomial random sampling for categorical variables. The follow-up data included three types of events: recurrence, death and last follow-up, where the recurrence and death were defined as the composite event of interest in the EFS analysis. The last follow-up occurred before the events of interest were considered as the censoring event. In this cohort, 164 recurrence events, 45 dead events, and 199 censoring events were observed.

### Analysis

**Fig. 1:**
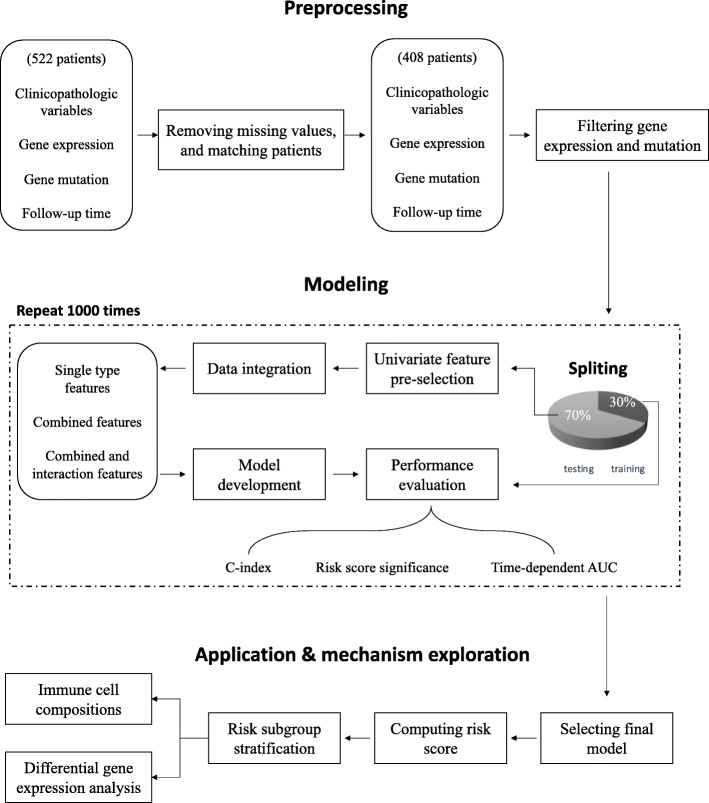
Workflow used in this study

As shown in Fig. 1, the workflow in this study can be sketched in four parts: (1) data preprocessing, (2) feature integration and model development using the training set, (3) prediction and model evaluation using the testing set, and (4) exploration of molecular mechanisms related to differential prognostic risk.

### Data preprocessing

We downloaded the gene expression and somatic mutation detection data generated by TCGA group [18] for this study. We used the fragments per kilobase of transcript per Million mapped reads (FPKM) value to represent gene expression level. We restricted our analysis to genes with a FPKM summed across all samples greater than 500 and with non-zero expression in at least 200 patients. These genes were further filtered based on variance, in which genes with a standard error of log-transformed FPKM across samples greater than 0.4 were retained. This resulted in a total of 7401 genes for model development. For gene mutation, we pooled single nucleotide variations (SNVs) and indels for analysis. We filtered out mutated genes, defined as those with at least one somatic mutation, occurred in fewer than 30 samples. A total of 271 genes passed such filtering. The distribution of the included clinicopathologic variables and genes with top 10 mutation frequencies were summarized in Table S1.

### Model development

We applied univariate Cox regression model for feature pre-selection of the gene expression or mutation data. The model was fitted between each feature and EFS, and the importance of feature was determined by their ward test *p* value. We set the *p* value (unadjusted for multiple testing) cutoff as 3e−04 and 0.08 for gene expression and mutation, respectively. We then used lasso Cox regression [19] to develop the predictive model, using the pre-selected features as the input.

For model development, we randomly split the study cohort into training (285 patients) and testing (123 patients) sets. The input data for prediction model were prepared in following ways (Fig. 1 and Table S2). In the first way, the features processed as described above were simply used as the input data without any further modification. In the second way, we used the univariate Cox model to narrow down the searching scope of gene expression and mutation data, respectively. These pre-selected genes were used as the input for prediction model development. In the third way, we performed a pairwise combination between the three types of predictors. We also included a combination that uses all three types of data as the input. Only the genes pre-selected in the second way were used here. In the fourth way, we added interaction terms to those prepared in the third way. The interaction features were generated by multiplication of any two or three types of features from the input. An interaction feature was included in predictive modeling only when its distribution is not severely unbalanced. A total of 14 different model input feature sets were constructed.

We used the one-standard-error rule [20] and the 3-fold cross validation method to find an optimized L1 penalty value for every input set using the training dataset. This penalty was finally used to develop a lasso Cox model using all training dataset.

### Model evaluation

We used the C-index [21] as one criteria to evaluate the predictive performance of models on the testing dataset. We performed 1000 repetitions of model development and evaluation to mitigate the bias caused by data splitting. The score *X*_test_*β* was computed by multiplying the model coefficients and the features in testing dataset. This newly generated variable was analyzed in the Cox models to evaluate its significance related to EFS. Furthermore, we calculated *Xβ* as the risk score to stratify patients within specifically defined subgroups. The Kaplan-Meier method was then used to analyze their event-free survival distribution.

We also used competing risks modeling to evaluate the significance of the risk score as a univariate predictor for RFS events. As sketched in Fig. 3a, after initial treatment, one may progressed to recurrence (from 1 to 2) or to death (from 1 to 3). The death event would stop patients from having a recurrence, and thus posed a competing risk to recurrence. The competing risks model applied the cumulative incidence function (CIF) *I*_*k*_(*t*) to calculating the cumulative probability of each cause. The computational formula of CIF is given by

$$ {I}_k(t)=\Pr \left(T\le t,D=k\right)={\int}_0^t{\lambda}_k(t)S(s) ds $$

where *λ*_*k*_(*t*) is the hazard of cause k at time t, $S(t)=\exp \left[-{\sum}_{k=1}^K{\int}_0^t{\lambda}_k(s) ds\right]$ is the survival function. To incorporate covariates information, we used Fine and Gray method [22] to impose a proportional hazards assumption on the subdistribution hazard:

$$ {\overline{\lambda}}_k\left(t|\mathbf{Z}\right)={\overline{\lambda}}_{k,0}(t)\exp \left({\boldsymbol{\beta}}_k^T\mathbf{Z}\right) $$

where ***β*** is a vector of coefficients and **Z** is a matrix of covariates. Individuals who fail from another cause are remained in the risk set for ${\overline{\lambda}}_k(t)$ estimation [23]. The time-dependent AUC [24] was computed to evaluate the model fitting. The confidence interval was computed based on Blanche et al. [25]. All analyses were performed by R version 3.6 and packages including survival, glmnet, caret, cmprsk, and riskRegression.

### Exploration of underlying mechanisms

For differential gene expression analysis, we used the negative binomial generalized linear model with tag-wise dispersion in R package edgeR [26]. The raw count data was normalized by the TMM (the trimmed mean of M values) [27]. Only genes whose mean of counts was more than 15 reads and with non-zero count in every sample were retained for normalization. This resulted in a total of 15,507 genes used for downstream analysis. We performed gene sets enrichment analyses using multiple algorithms including GOseq, Enrichr, and GSEA [28–30].

We used CIBERSORT software to deconvolve the relative fractions of different immune cell types from the RNA sequencing data [31]. To infer the significance of enrichment of cell types between the higher and lower-risk patient subgroups, we used Wilcoxon rank-sum test to compute *p* values. All *p* values were corrected by Benjamini-Hochberg procedure to control the false discovery rate (FDR) and to obtain the adjusted *p* values [32].

## Result

### Patient characteristics and feature processing

The study workflow was sketched in Fig. 1. We enrolled a total of 408 patients with complete information in EFS data, major relevant clinicopathologic variables, and gene expression and mutation profiles. The median EFS time was 809 days (Figure S1; 95% CI 692, 1018). We randomly split the data into model development dataset (285 patients) and testing dataset (123 patients) with a comparable censoring ratio (48.9% vs. 48.6%) for 1000 times. The average median EFS time for the development and testing dataset were 822 and 834 days, respectively. The distribution of included clinicopathologic variables of the cohort was summarized in Table S1. Only AJCC tumor stage and adjuvant treatment were significantly associated with EFS.

For model input data integration, we prepared 5 sets of features selected from single type of data (single type features), 4 sets of features combined from different types of data (combined features), and 5 sets of features incorporated with interaction terms generated within (intra-type) each type of data or between (inter-type) different types of data (combined and interaction features). The size of each feature space was summarized in Table S2.

### Comparison of integrated prognostic models

We first compared the prediction accuracy of models developed based on single type of input features. The performance of models based on clinicopathologic variables was the best with a C-index of 0.624 ± 0.028 on the testing set (Figure S2A and Table S3). To assess the effectiveness of feature pre-selection, we compared models using features with or without univariate Cox analysis. For gene expression, the mean of testing C-index increased by 0.029 with univariate pre-selection (*p* value < 0.001), while for mutation profiles, the mean of prediction accuracy increased by 0.013 (*p* value < 0.001). These indicated the benefit of feature pre-selection step.

We next compared prognostic models that integrate different types of input data. The best prediction model was the model combining three types of input data, which achieved a significantly higher mean C-index (0.639 ± 0.033) on the testing data as compared to the clinicopathologic model (*p* value < 0.001; Figure S2B and Table S3). We then assessed whether the inter-type and intra-type interaction covariates can improve the prediction accuracy. Adding interaction covariates had limited benefits on prediction power (Figure S3C and Table S3). The final data integration we presented in this study was thus the one combining three types input variables without interactions.

### Assessment of significance of the prognostic risk score

A successful application of a prognostic model requires a risk score that can be readily computed for clinical use. We therefore selected an individual model with a C-index (0.638) close to the mean of final data integration as described above, and calculated the linear combination of coefficients and features from the model as the event-risk score (or mathematically X*β*). We named this score as the mul-score (Table S4).

To further evaluate the significance of mul-score as compared to that of the cln-score (the risk score computed by clinicopathologic variables-based model), we fitted Cox proportional hazard models by setting the score as the single covariate on the testing set. For a fair comparison, we computed the cln-score from a model with a C-index (0.622) also close to the mean of clinicopathologic variables-based data integration. The *p* value of mul-score coefficient was more significant than that of cln-score in such univariate modeling (Table S5). When a multivariate Cox model was fitted using the two scores as covariates, only the mul-score was still statistically significant (Table S5). This suggested that the mul-score could capture extra EFS-related information that was not considered by cln-score.

**Fig. 2:**
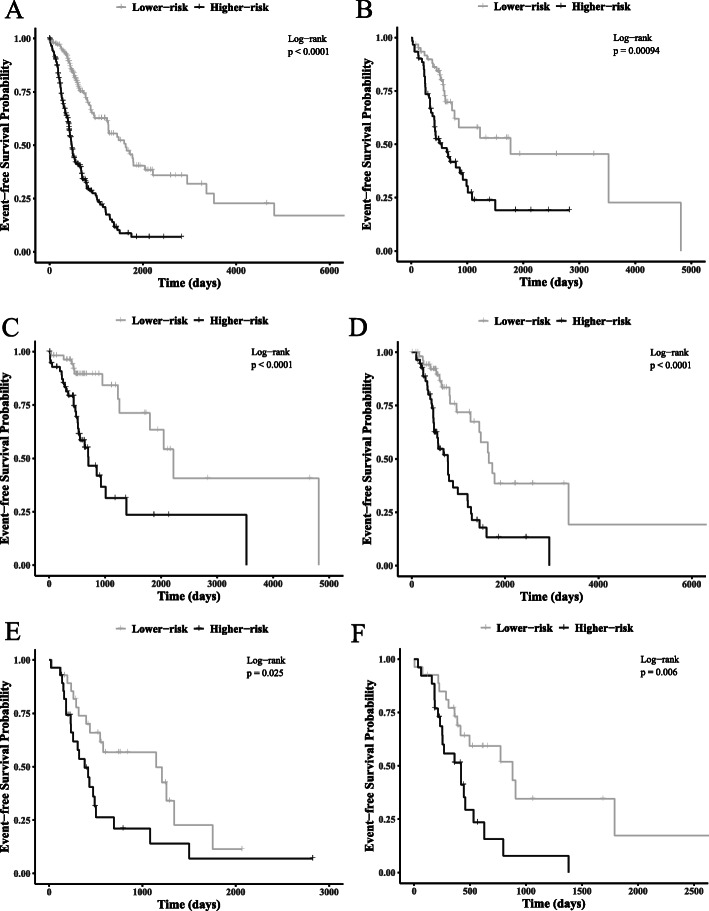
Kaplan-Meier curve of higher-risk and lower-risk subgroups as stratified by mul-score within different groups of patients. The sets from **a** to **f** are: **a**, all patients; **b**, testing set; **c**, patients in AJCC pathologic tumor stage IA; **d**, patients in AJCC pathologic tumor stage IB; **e**, patients in AJCC pathologic tumor stage IIB; **f**, patients in AJCC pathologic tumor stage III

We then investigated the risk stratification effectiveness of the two risk scores within specific groups of patients (Table S6). We found that the mul-score was not only significantly associated with EFS in each group, but also showed a higher level of relevance than that of the cln-score, as reflected by the fitting *p* values. We set the score median within each group as the threshold to stratify for the higher- and lower-risk subgroups. The mul-score generated a more striking stratification within each set of patients as compared to the cln-score (Fig. 2, Figure S3, Table S6). For example, for stage IA subgroup, the mul-score identified a higher-risk subgroup with a median EFS time 18 months earlier than that of the cln-score (702 vs. 1255 days). On the other hand, for stage IIB, the mul-score revealed a significantly lower-risk subgroup who would develop an event 19 months later than that of the cln-score (1146 vs. 578 days).

### RFS analysis

**Fig. 3:**
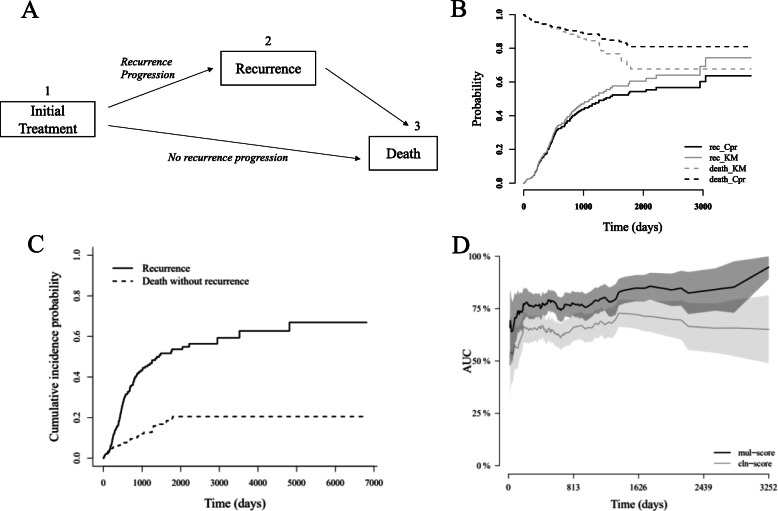
RFS analysis results. **a** Sketch of disease process for patients after initial treatment. **b** Estimated cumulative probabilities for recurrence and estimated survival (1, cumulative probability) for death without recurrence, using either competing risks modeling (Cpr) or naïve KM modeling (KM). **c** The cumulative probability of developing recurrence and death without recurrence. **d** Comparison of time-dependent AUCs of risk scores for RFS prediction

We next assessed the significance of proposed risk score as a predictor for RFS. We used competing risks models for the assessment because a total of 45 death events without recurrence observed in the cohort can act as the competing event for recurrence risk analysis (Fig. 3a). Ignoring the effect from such competing event could lead to an over-estimation of recurrence risk (Fig. 3b) since those who died without recurrence were still considered as having possibility of developing recurrence. The cumulative probabilities of these two types of events were shown in Fig. 3c. Most failure events of both causes occurred before about day 2000, and the failure rate became lower after then for recurrence while unchanged for direct death. The mean time-dependent AUC of mul-score for RFS prediction was significantly higher than that of the cln-score (Fig. 3d; 0.772 vs. 0.646, *p* value < 0.001).

### Differential risk-related immune phenotyping variations

To explore the variations of immune phenotyping associated with differential prognostic risk, we performed differential gene expression analysis between the higher-risk (*n* = 204) and lower-risk (*n* = 204) patients as determined by mul-score. A total of 7250 genes was differentially expressed, and 2 GOs were identified as significantly enriched: extracellular matrix organization (GO 0030198, adjusted *p* value < 0.001) and extracellular structure organization (GO 0043062, adjusted *p* value = 0.001).

For the immune phenotyping analysis, a total of 519 differentially expressed genes described above was immune-related according to a curated list generated from the immunology database and analysis portal (ImmPort) [33]. We further identified that five of them were also on the list of differentially expressed genes detected within stage IIB and within IIIA subgroups (Fig. 2 and Table S7). As expected, the proto-oncogenes *PTPN1*, *JAK1*, *JAG1* [34–36] were significantly upregulated in the higher-risk patients. However, another gene *NENF*, previously being reported as promoting cancer development [37], was downregulated, suggesting the complexity of tumor immune microenvironment in a high prognostic risk scenario.

We then used computational deconvolution methods to investigate the variations of immune cell compositions between the two risk subgroups. As shown in Figure S4, the inferred relative fractions of immune cell compositions varied both within and across risk subgroups (Figure S4A). In higher-risk patients, we observed a significant depletion of mast cells and activated natural killer cells (Figure S4B), indicating a transformation of innate immunity in LUAD tumor microenvironment from activating to suppressive status.

## Discussion

Our study developed and validated a new prognostic model integrating clinicopathologic variables, gene expression, and mutation data as the input. We used testing datasets to show that the model achieved a higher level of accuracy of EFS prediction than models based on any other input data integrations. Adding interaction covariates to prognostic models showed limited benefits on improving prediction power. We further compared at the level of risk score computed from these models. The univariate model fitting *p* values of the score indicated that the one generated from the best combinatorial model captured a wider spectrum of EFS-related variations. Moreover, the proposed score provided a higher-resolution EFS stratification for pathologically defined subgroup of patients and showed superior time-varying RFS prediction power than conventional clinicopathologic methods (mean time-dependent AUC = 0.772 vs. 0.646, *p* value < 0.001). The higher-risk subgroup determined by the score was characterized with RNA expression aberration of multiple immune-associated genes and depletion of activated natural killer cells and mast cells.

Integrating different types of data is an effective way to improve prognostic prediction. In this study, the model integrating clinicopathologic variables, gene expression, and mutation achieved the best performance in multiple evaluation metrics. Our conclusions were consistent with previous studies. For example, Chen et al. [13] integrated two micro-RNAs, two mRNAs, and two DNA methylation sites as prognostic factors associated with OS, and they achieved a more significant risk stratification within pathologically defined subgroups. Song et.al showed that, by integrating genetic mutations and expression profiles with clinicopathologic variables, the prediction of both OS and RFS showed the highest cross-validation accuracy among all the models in the TCGA-LUAD data [15]. Besides, Dong et al. [14] found that by adding DNA methylation and gene expression biomarkers to a model using only clinical data as the input, the AUCs improved by 18.3% and 16.4% in discovery and validation phases for early-stage LUAD patients, respectively. We extended some of these studies by introducing more types of input data integrations and stricter evaluation criteria. The resultant model not only showed improved prediction for EFS on the testing dataset, but also demonstrated its significance as a predictor for RFS under a bias-corrected competing risks modeling framework.

Our study also suggested new therapeutic opportunities for the higher-risk patients. For example, we discovered that the exhaustion of innate immunity components was correlated with prognostic risk. The natural killer cells are lymphocytes that can recognize transformed cells via surface receptor interaction. It has been shown in a recent publication [38] that the activation of natural killer cells promotes the efficacy of LUAD immunotherapy in mouse model by enhancing the adaptive immune responses. The abundance of mast cells was shown to be positively correlated with the survival of early-stage LUAD patients, and mast cells-related gene signatures can be used for predicting survival probabilities [39]. Moreover, it has been reported that the interaction between mast cells and natural killer cells is critical for anti-viral defense [40]. Whether there exist similar interactions important for anti-LUAD effect warrants experimental investigations.

Our proposed score included the expression profile of 13 genes and somatic mutation profile of 10 genes. We recognized that this is a relatively large panel of testing which involves both expression and mutation measurements. However, there already exists multi-panel testing technologies that can be readily translated for the score. For example, a 21-gene expression panel (Oncotype DX) based on qRT-PCR platform has already been made for clinical use to inform breast cancer treatment [41]. For mutational testing, a 324 gene panel (FoundationOne CDx )[42] based on next-generation sequencing (NGS) platform was approved for clinical genetic testing by FDA recently. We thus think the score has potential to be cost-effective with these multi-panel testing technologies.

Our study has limitations. First, the combinatorial models developed in this study were based on features already selected by models based on individual type of input data, using the same training dataset. This introduced more overfitting and could possibly cause the failure of selecting truly important combinatorial models. Second, the feature pre-selection method remains to be improved. We performed univariate Cox analysis to pre-select important features, and this method only provided a slight improvement for models based on single-type variables. Third, the EFS outcome we defined in this study included death from any causes. We recognized that including death not related to lung cancer could bias EFS estimates, but such detail information was not available from TCGA clinical dataset. We mitigated this by further evaluating the score on RFS analysis. Fourth, all analyses were performed on TCGA-LUAD dataset. More external validations should be made before considering clinical translation of the score.

## Conclusions

In summary, our study proposed a novel prognostic risk score integrating clinicopathologic variables, gene expression, and mutation data for LUAD. The score was useful for both EFS and RFS analyses.

## Supplementary information

**Additional file 1: Table S1**: Summary of distribution and EFS association of clinicopathologic variables and genes with top 10 mutation frequencies used in the study. **Table S2**: Description of input feature sets and the size of feature space. **Table S3**: The summary of C-index, related to Figure S2. **Table S4**: The name and coefficient of features of the selected combinatorial model. **Table S5**: Summary of p values of the coefficients in models fitted with single or multiple risk scores on the testing datasets. **Table S6**: Summary of model fitting results (likelihood ratio test) and median survival time (in days) of higher- and lower-risk subgroups within each sets of patients. **Table S7**: Summary of 5 immune-related genes commonly identified as differentially expressed within all patients, stage IIB and IIIA subgroups. **Table S8**: List of the TCGA ID for the higher or lower-risk subgroup as stratified by our proposed score. **Figure S1**: The Kaplan-Meier curve for EFS of 408 patients enrolled in this study. **Figure S2**: The C-index of models developed based on single type of data (A) combined feature sets (B) and models with interaction covariates (C). The white box summarized C-index for models selected from cross-validation (1 standard error rule), while the gray box for those from testing. Abbreviations can be referred to Table S2. **Figure S3**: Kaplan-Meier curve of higher- and lower-risk subgroups stratified by cln-score within different sets of patients. The sets from A to F are: A, all patients; B, testing set; C, patients in AJCC pathologic tumor stage IA; D, patients in AJCC pathologic tumor stage IB; E, patients in AJCC pathologic tumor stage IIB; F, patients in AJCC pathologic tumor stage IIIA. **Figure S4**: Barplot summary of inferred relative fractions of cell types (A) and volcano plot summary for the significance of difference in immune cellular compositions between the higher and lower-risk subgroup patients (B).

## Data Availability

The datasets analyzed for the current study are available in the TCGA-LUAD repository: https://portal.gdc.cancer.gov/projects/TCGA-LUAD.

## Abbreviations

LUAD: Lung adenocarcinoma
TCGA: The Cancer Genome Atlas
AJCC: American Joint Committee on Cancer
EFS: Event-free survival
OS: Overall survival
RFS: Recurrence-free survival
FPKM: Fragments per kilobase of transcript per million mapped reads
CIF: Cumulative incidence function
mul-score: The risk score computed by the best combinatorial model
cln-score: The risk score computed by clinicopathologic variables-based model
Lasso: Least absolute shrinkage and selection operator
I2: The interaction features generated by multiplication of any two features within each type of data or between different types of data;
I3, I2: The interaction features generated by multiplication of any three features between different types of data
