# Supplementary material for: Development of a novel prognostic score combining clinicopathologic variables, gene expression, and mutation profiles for lung adenocarcinoma
Source: World J Surg Oncol. 2020 Sep 19;18:249. doi: 10.1186/s12957-020-02025-0 (PMC7502202; doi:10.1186/s12957-020-02025-0)
Supplement: Supplementary file 1 — Additional file 1: Table S1: Summary of distribution and EFS association of clinicopathologic variables and genes with top 10 mutation frequencies used in the study. Table S2: Description of input feature sets and the size of feature space. Table S3: The summary of C-index, related to Figure S2. Table S4: The name and coefficient of features of the selected combinatorial model. Table S5: Summary of p values of the coefficients in models fitted with single or multiple risk scores on the testing datasets. Table S6: Summary of model fitting results (likelihood ratio test) and median survival time (in days) of higher- and lower-risk subgroups within each sets of patients. Table S7: Summary of 5 immune-related genes commonly identified as differentially expressed within all patients, stage IIB and IIIA subgroups. Table S8: List of the TCGA ID for the higher or lower-risk subgroup as stratified by our proposed score. Figure S1: The Kaplan-Meier curve for EFS of 408 patients enrolled in this study. Figure S2: The C-index of models developed based on single type of data (A) combined feature sets (B) and models with interaction covariates (C). The white box summarized C-index for models selected from cross-validation (1 standard error rule), while the gray box for those from testing. Abbreviations can be referred to Table S2. Figure S3: Kaplan-Meier curve of higher- and lower-risk subgroups stratified by cln-score within different sets of patients. The sets from A to F are: A, all patients; B, testing set; C, patients in AJCC pathologic tumor stage IA; D, patients in AJCC pathologic tumor stage IB; E, patients in AJCC pathologic tumor stage IIB; F, patients in AJCC pathologic tumor stage IIIA. Figure S4: Barplot summary of inferred relative fractions of cell types (A) and volcano plot summary for the significance of difference in immune cellular compositions between the higher and lower-risk subgroup patients (B). [file 12957_2020_2025_MOESM1_ESM.docx]

**Supplementary materials**

**Development of a novel prognostic score combining clinicopathologic variables, gene expression and mutation profiles for lung adenocarcinoma**

**Guofeng Li^1^, Guangsuo Wang^1^, Yanhua Guo^2^, Shixuan Li^1^, Youlong Zhang^3^, Jialu Li^3^*, Bin Peng^1^***

1 Department of Thoracic Surgery, Shenzhen People's Hospital, Second Clinical Medical College of Jinan University

2 Department of Thoracic Surgery, Shanghai Pulmonary Hospital, School of Medicine, Tongji University

3 Department of Biostatistics, HuaJia Biomedical Intelligence

*Correspondence to:*

Jialu Li, PhD, Department of Biostatistics, HuaJia Biomedical Intelligence, Shenzhen Overseas Chinese High-Tech Venture Park, Nanshan district, Shenzhen, China, 518057. Email: [Jialu.li@huajiabio.com](mailto:Jialu.li@huajiabio.com)

Or to

Bin Peng, MD, Department of Thoracic Surgery, Shenzhen People's Hospital, Luohu district, Shenzhen, China, 518020. Email: 183672297@qq.com

Contents

[Supplementary Tables 3](#_Toc38040508)

[Supplementary Figures 9](#_Toc38040509)

# Supplementary Tables

Table S1: Summary of distribution and EFS association of clinicopathologic variables and genes with top 10 mutation frequencies used in the study.

| **Variables** | **Distribution** | **p-value** |
| --- | --- | --- |
| Tobacco smoking status | 408 (100%) | / |
| lifelong non-smoker | 64 (15.7%) | 0.324 |
| current smoker | 99 (24.3%) | 0.137 |
| current reformed smoker | 245 (60%) | 0.512 |
| AJCC pathologic tumor stage | 408 (100%) | <0.001 |
| stage I | 229 (56.1%) | / |
| stage II, III, IV | 179 (43.9%) | / |
| Adjuvant radiation treatment | 408 (100%) | <0.001 |
| yes | 52 (12.7%) | / |
| no | 356 (87.3%) | / |
| Adjuvant pharmaceutical treatment | 408 (100%) | 0.022 |
| yes | 137 (33.6%) | / |
| no | 271 (66.4%) | / |
| Age at diagnosis | 398 (97.5%) | 0.091 |
| mean/std | 65.1/10.2 | / |
| Gender | 408 (100%) | 0.652 |
| male | 181 (44.4%) | / |
| female | 227 (55.6%) | / |
| History of other malignancies | 408 (100%) | 0.382 |
| yes | 77 (18.9%) | / |
| no | 331 (81.1%) | / |
| Anatomic organ subdivision | 400 (98%) | / |
| left lower | 59 (14.8%) | 0.576 |
| left upper | 95 (23.8%) | 0.393 |
| right lower and middle | 90 (22.5%) | 0.760 |
| right upper | 153 (38.2%) | 0.430 |
| TP53 | 199 (48.8%) | 0.563 |
| TTN | 189 (46.3%) | 0.491 |
| MUC16 | 163 (40%) | 0.771 |
| CSMD3 | 158 (38.7%) | 0.179 |
| RYR2 | 150 (36.8%) | 0.445 |
| LRP1B | 132 (32.4%) | 0.780 |
| ZFHX4 | 127 (31.1%) | 0.648 |
| USH2A | 126 (30.9%) | 0.965 |
| KRAS | 110 (27%) | 0.779 |
| FLG | 101 (24.8%) | 0.128 |

Table S2: Description of input feature sets and the size of feature space.

| Items | Feature sets | Mean number |
| --- | --- | --- |
| single type features | clinicopathologic variables (cln.) | 8 |
|  | gene expression after pre-filtering (expf.) | 7401 |
|  | gene mutation after pre-filtering (mutf.) | 271 |
|  | gene expression after univariate feature pre-selection (exps.) | 23 |
|  | gene mutation after univariate feature pre-selection (muts.) | 22 |
| combined features | clinicopathologic variables and pre-selected gene expression (cln. & exps.) | 31 |
|  | clinicopathologic variables and pre-selected gene mutation (cln. & muts.) | 30 |
|  | Pre-selected gene expression and mutation (exps. & muts.) | 45 |
|  | clinicopathologic variables, pre-selected gene expression and mutation (cln. & exps. & muts.) | 53 |
| combined and interaction features | clinicopathologic variables and pre-selected gene expression, with I2 (cln. : exps.) | ~496 |
|  | clinicopathologic variables and pre-selected gene mutation, with I2 (cln. : muts.) | ~485 |
|  | pre-selected gene expression and mutation, with I2 (exps. : muts.) | ~945 |
|  | clinicopathologic variables, pre-selected gene expression and mutation, with I2 (cln. : exps. : muts.) | ~1431 |
|  | clinicopathologic variables, pre-selected gene expression and mutation, with I2 and I3  (cln. :: exps. :: muts.) | ~4100 |

The “&” represents combination; the “:” represents the intra-type and inter-type interaction; the “::” indicates adding the inter-type interaction of three types of data to “:”; the “~” represents that there might be a slight change on the counts because of unbalanced variables generated by the interaction. I2, the interaction features generated by multiplication of any two features within each type of data or between different types of data; I3, I2 and the interaction features generated by multiplication of any three features between different types of data.

Table S3: The summary of C-index, related to Figure S2.

| Feature sets | cv1se | | Testing | |
| --- | --- | --- | --- | --- |
|  | Mean | Standard error | Mean | Standard error |
| cln. | 0.628 | 0.019 | 0.624 | 0.028 |
| expf. | 0.564 | 0.029 | 0.549 | 0.039 |
| mutf. | 0.551 | 0.032 | 0.537 | 0.037 |
| exps. | 0.634 | 0.021 | 0.578 | 0.035 |
| muts. | 0.649 | 0.038 | 0.550 | 0.038 |
| cln.&exps. | 0.679 | 0.02 | 0.633 | 0.031 |
| cln.&muts. | 0.686 | 0.022 | 0.631 | 0.033 |
| exps.&muts. | 0.69 | 0.025 | 0.588 | 0.037 |
| cln.&exps.&muts. | **0.714** | 0.022 | **0.639** | 0.033 |
| cln.:exps. | 0.675 | 0.02 | 0.624 | 0.032 |
| cln.:muts. | 0.686 | 0.023 | 0.633 | 0.032 |
| exps.:muts. | 0.682 | 0.025 | 0.582 | 0.037 |
| cln.:exps.:muts. | 0.705 | 0.023 | 0.627 | 0.035 |
| cln.::exps.::muts. | 0.704 | 0.024 | 0.628 | 0.033 |

Table S4: The name and coefficient of features of the selected combinatorial model.

| Feature types | Feature names | Coefficients |
| --- | --- | --- |
| gene  expression | UBXN11 | -0.023 |
|  | AL691432.2 | -0.157 |
|  | CES4A | -0.080 |
|  | SGPP1 | 0.355 |
|  | FRMD6 | 0.067 |
|  | CD109 | 0.045 |
|  | ARFGAP1 | -0.127 |
|  | DKK1 | 0.087 |
|  | AC025181.2 | -0.149 |
|  | ZMYND12 | -0.099 |
|  | ARMH1 | -0.083 |
|  | VEGFC | 0.041 |
|  | CCL20 | 0.088 |
| gene  mutation | AHNAK | 0.087 |
|  | BIRC6 | 0.205 |
|  | PKHD1 | -0.024 |
|  | ABCA13 | -0.069 |
|  | CTNNA2 | -0.190 |
|  | DNAH5 | -0.043 |
|  | ZNF521 | 0.167 |
|  | CSMD3 | -0.124 |
|  | EPHA3 | -0.186 |
|  | KMT2C | 0.209 |
| clinicopathologic  variables | tobacco_smoking_history_indicator.2^*^ | -0.176 |
|  | ajcc_pathologic_tumor_stage^&^ | 0.432 |
|  | radiation_treatment_adjuvant | 0.388 |

^*^The tobacco_smoking_history_indicator.2 is a binary variable with 1 for current smoker and 0 others;

^&^The ajcc_pathologic_tumor_stage is a binary variable with 1 for stage≥III and 0 for stage<III.

Table S5: Summary of p values of the coefficients in models fitted with single or multiple risk scores on the testing datasets.

| Model types | cln-score | mul-score |
| --- | --- | --- |
| univariate Cox | 0.0007 | 0.0003 |
| multivariate Cox | 0.1697 | 0.0478 |

Table S6: Summary of model fitting results (likelihood ratio test) and median survival time (in days) of higher- and lower-risk subgroups within each sets of patients.

| Risk scores | Terms | All (209/408) | Testing set (62/123) | Stage IA (39/114) | Stage IB (53/110) | Stage IIB (37/56) | Stage IIIA (34/53) |
| --- | --- | --- | --- | --- | --- | --- | --- |
| cln-score | LR | 42.23 | 10.7 | 0.91 | 5.18 | 3.27 | 5.28 |
|  | LR p | 8.00E-11 | 0.001 | 0.3 | 0.02 | 0.07 | 0.02 |
|  | Lower-risk | 1447  (1194, 1798) | 1773  (1009, NA) | 1798  (950, NA) | 1268  (883, 1653) | 578  (395, 1340) | 772  (419, NA) |
|  | Higher-risk | 497  (441, 688) | 502  (379, 772) | 1255  (697, NA) | 772  (478, NA) | 396  (305, NA) | 414  (231, 795) |
| mul-score | LR | 119.7 | 12.81 | 15.23 | 22.38 | 12.3 | 11.74 |
|  | LR p | <2e-16 | 3.00E-04 | 1.00E-04 | 2.00E-06 | 5.00E-04 | 6.00E-04 |
|  | Lower-risk | 1632  (1258, 2218) | 1773  (772, NA) | 2218  (1798, NA) | 1653  (1447, NA) | 1146  (545, NA) | 879  (414, NA) |
|  | Higher-risk | 477  (424, 568) | 545  (414, 987) | 702  (524, NA) | 772  (545, 1194) | 379  (258, 692) | 418  (252, 625) |

The LR and LR p represent likelihood ratio test statistic and its p value, respectively. The Stage represents the AJCC pathologic tumor stage. The number of events and patients were summarized like “(209/408)” in the first row. The 95% confidence interval of median survival time was summarized like “(1194, 1798)”. We only analyzed the pathology-defined sets with a sample size greater than 50.

Table S7: Summary of 5 immune-related genes commonly identified as differentially expressed within all patients, stage IIB and IIIA subgroups.

| **Symbol** | **EntrezID** | **logFC** | **logCPM** | **FDR p-value** |
| --- | --- | --- | --- | --- |
| PTPN11 | 5781 | 0.3225 | 6.978 | 5.48E-08 |
| JAK1 | 3716 | 0.2342 | 7.546 | 2.53E-05 |
| JAG1 | 182 | 0.3935 | 5.861 | 0.0007 |
| UBR1 | 197131 | 0.1595 | 5.444 | 0.0078 |
| NENF | 29937 | -0.1862 | 6.094 | 0.0195 |

Table S8: List of the TCGA ID for the higher or lower-risk subgroup as stratified by our proposed score.

| TCGA ID | risk stratification |
| --- | --- |
| TCGA-NJ-A4YF | Lower-risk |
| TCGA-71-6725 | Lower-risk |
| TCGA-95-7948 | Lower-risk |
| TCGA-78-8640 | Lower-risk |
| TCGA-49-AARR | Lower-risk |
| TCGA-49-AAR0 | Lower-risk |
| TCGA-69-7979 | Lower-risk |
| TCGA-49-AARQ | Lower-risk |
| TCGA-97-8552 | Lower-risk |
| TCGA-MP-A4T6 | Lower-risk |
| TCGA-44-5644 | Lower-risk |
| TCGA-78-8662 | Lower-risk |
| TCGA-NJ-A55R | Lower-risk |
| TCGA-55-8097 | Lower-risk |
| TCGA-MN-A4N1 | Lower-risk |
| TCGA-55-8087 | Lower-risk |
| TCGA-55-6972 | Lower-risk |
| TCGA-49-AAR2 | Lower-risk |
| TCGA-L9-A8F4 | Lower-risk |
| TCGA-O1-A52J | Lower-risk |
| TCGA-NJ-A55A | Lower-risk |
| TCGA-97-A4M1 | Lower-risk |
| TCGA-91-6835 | Lower-risk |
| TCGA-55-8512 | Lower-risk |
| TCGA-75-7025 | Lower-risk |
| TCGA-49-AARE | Lower-risk |
| TCGA-55-A492 | Lower-risk |
| TCGA-NJ-A4YQ | Lower-risk |
| TCGA-99-AA5R | Lower-risk |
| TCGA-97-A4M2 | Lower-risk |
| TCGA-86-8358 | Lower-risk |
| TCGA-78-7535 | Lower-risk |
| TCGA-64-5778 | Lower-risk |
| TCGA-55-6986 | Lower-risk |
| TCGA-L4-A4E5 | Lower-risk |
| TCGA-S2-AA1A | Lower-risk |
| TCGA-44-6148 | Lower-risk |
| TCGA-44-8117 | Lower-risk |
| TCGA-97-7937 | Lower-risk |
| TCGA-55-8206 | Lower-risk |
| TCGA-91-8499 | Lower-risk |
| TCGA-55-A4DG | Lower-risk |
| TCGA-93-A4JO | Lower-risk |
| TCGA-55-7570 | Lower-risk |
| TCGA-L9-A7SV | Lower-risk |
| TCGA-62-A46P | Lower-risk |
| TCGA-78-7163 | Lower-risk |
| TCGA-86-8073 | Lower-risk |
| TCGA-86-8673 | Lower-risk |
| TCGA-NJ-A4YG | Lower-risk |
| TCGA-44-7660 | Lower-risk |
| TCGA-44-A47A | Lower-risk |
| TCGA-55-1592 | Lower-risk |
| TCGA-55-1596 | Lower-risk |
| TCGA-78-7147 | Lower-risk |
| TCGA-44-A4SU | Lower-risk |
| TCGA-J2-A4AE | Lower-risk |
| TCGA-91-6836 | Lower-risk |
| TCGA-86-8585 | Lower-risk |
| TCGA-97-A4M7 | Lower-risk |
| TCGA-38-4630 | Lower-risk |
| TCGA-44-5645 | Lower-risk |
| TCGA-55-8514 | Lower-risk |
| TCGA-55-8302 | Lower-risk |
| TCGA-55-8301 | Lower-risk |
| TCGA-55-6985 | Lower-risk |
| TCGA-73-7498 | Lower-risk |
| TCGA-99-8028 | Lower-risk |
| TCGA-55-8510 | Lower-risk |
| TCGA-95-7039 | Lower-risk |
| TCGA-75-5147 | Lower-risk |
| TCGA-MP-A4TA | Lower-risk |
| TCGA-05-4405 | Lower-risk |
| TCGA-62-A46S | Lower-risk |
| TCGA-44-2657 | Lower-risk |
| TCGA-55-8203 | Lower-risk |
| TCGA-55-8507 | Lower-risk |
| TCGA-L9-A444 | Lower-risk |
| TCGA-62-A46V | Lower-risk |
| TCGA-55-7576 | Lower-risk |
| TCGA-05-4420 | Lower-risk |
| TCGA-49-4486 | Lower-risk |
| TCGA-55-7573 | Lower-risk |
| TCGA-69-8255 | Lower-risk |
| TCGA-91-8497 | Lower-risk |
| TCGA-MN-A4N5 | Lower-risk |
| TCGA-97-8171 | Lower-risk |
| TCGA-55-6980 | Lower-risk |
| TCGA-86-A4P7 | Lower-risk |
| TCGA-73-7499 | Lower-risk |
| TCGA-55-7728 | Lower-risk |
| TCGA-67-6215 | Lower-risk |
| TCGA-91-8496 | Lower-risk |
| TCGA-64-1676 | Lower-risk |
| TCGA-44-6147 | Lower-risk |
| TCGA-97-A4M0 | Lower-risk |
| TCGA-75-6206 | Lower-risk |
| TCGA-L4-A4E6 | Lower-risk |
| TCGA-91-6831 | Lower-risk |
| TCGA-69-A59K | Lower-risk |
| TCGA-50-8457 | Lower-risk |
| TCGA-44-6144 | Lower-risk |
| TCGA-86-7954 | Lower-risk |
| TCGA-44-7667 | Lower-risk |
| TCGA-44-6776 | Lower-risk |
| TCGA-55-8621 | Lower-risk |
| TCGA-97-8172 | Lower-risk |
| TCGA-97-8179 | Lower-risk |
| TCGA-99-8025 | Lower-risk |
| TCGA-55-8508 | Lower-risk |
| TCGA-69-7980 | Lower-risk |
| TCGA-78-7162 | Lower-risk |
| TCGA-62-A46R | Lower-risk |
| TCGA-86-7955 | Lower-risk |
| TCGA-80-5608 | Lower-risk |
| TCGA-91-6828 | Lower-risk |
| TCGA-55-8207 | Lower-risk |
| TCGA-J2-A4AG | Lower-risk |
| TCGA-75-5146 | Lower-risk |
| TCGA-MP-A4TH | Lower-risk |
| TCGA-55-7903 | Lower-risk |
| TCGA-80-5611 | Lower-risk |
| TCGA-49-4514 | Lower-risk |
| TCGA-91-6829 | Lower-risk |
| TCGA-62-8399 | Lower-risk |
| TCGA-44-7669 | Lower-risk |
| TCGA-97-7941 | Lower-risk |
| TCGA-97-8177 | Lower-risk |
| TCGA-67-6217 | Lower-risk |
| TCGA-44-6778 | Lower-risk |
| TCGA-95-7944 | Lower-risk |
| TCGA-50-6594 | Lower-risk |
| TCGA-91-6840 | Lower-risk |
| TCGA-62-8397 | Lower-risk |
| TCGA-97-7546 | Lower-risk |
| TCGA-55-A491 | Lower-risk |
| TCGA-50-6597 | Lower-risk |
| TCGA-97-7553 | Lower-risk |
| TCGA-49-AARO | Lower-risk |
| TCGA-55-7913 | Lower-risk |
| TCGA-55-8092 | Lower-risk |
| TCGA-67-6216 | Lower-risk |
| TCGA-86-8669 | Lower-risk |
| TCGA-NJ-A7XG | Lower-risk |
| TCGA-55-8620 | Lower-risk |
| TCGA-05-4249 | Lower-risk |
| TCGA-55-A494 | Lower-risk |
| TCGA-69-7764 | Lower-risk |
| TCGA-86-8076 | Lower-risk |
| TCGA-55-A57B | Lower-risk |
| TCGA-91-6847 | Lower-risk |
| TCGA-97-7938 | Lower-risk |
| TCGA-86-8054 | Lower-risk |
| TCGA-55-8208 | Lower-risk |
| TCGA-44-8120 | Lower-risk |
| TCGA-86-8668 | Lower-risk |
| TCGA-44-7671 | Lower-risk |
| TCGA-95-8494 | Lower-risk |
| TCGA-44-A4SS | Lower-risk |
| TCGA-86-7953 | Lower-risk |
| TCGA-97-A4M5 | Lower-risk |
| TCGA-64-1681 | Lower-risk |
| TCGA-93-7348 | Lower-risk |
| TCGA-L9-A743 | Lower-risk |
| TCGA-86-8280 | Lower-risk |
| TCGA-44-3917 | Lower-risk |
| TCGA-55-8614 | Lower-risk |
| TCGA-86-6851 | Lower-risk |
| TCGA-75-7027 | Lower-risk |
| TCGA-55-8096 | Lower-risk |
| TCGA-97-A4M6 | Lower-risk |
| TCGA-55-8094 | Lower-risk |
| TCGA-97-A4LX | Lower-risk |
| TCGA-44-2661 | Lower-risk |
| TCGA-J2-A4AD | Lower-risk |
| TCGA-55-8204 | Lower-risk |
| TCGA-55-7995 | Lower-risk |
| TCGA-MN-A4N4 | Lower-risk |
| TCGA-55-7281 | Lower-risk |
| TCGA-73-4658 | Lower-risk |
| TCGA-78-7167 | Lower-risk |
| TCGA-73-4662 | Lower-risk |
| TCGA-55-8511 | Lower-risk |
| TCGA-95-7947 | Lower-risk |
| TCGA-L9-A50W | Lower-risk |
| TCGA-38-A44F | Lower-risk |
| TCGA-38-4631 | Lower-risk |
| TCGA-49-4512 | Lower-risk |
| TCGA-62-A471 | Lower-risk |
| TCGA-50-5931 | Lower-risk |
| TCGA-44-7662 | Lower-risk |
| TCGA-49-4501 | Lower-risk |
| TCGA-55-8513 | Lower-risk |
| TCGA-49-6745 | Lower-risk |
| TCGA-97-7547 | Lower-risk |
| TCGA-55-A48X | Lower-risk |
| TCGA-55-6978 | Lower-risk |
| TCGA-95-8039 | Lower-risk |
| TCGA-55-6543 | Lower-risk |
| TCGA-49-4488 | Lower-risk |
| TCGA-91-A4BD | Lower-risk |
| TCGA-05-4389 | Lower-risk |
| TCGA-49-AAR4 | Lower-risk |
| TCGA-44-A47B | Lower-risk |
| TCGA-55-8091 | Higher-risk |
| TCGA-44-7672 | Higher-risk |
| TCGA-MP-A5C7 | Higher-risk |
| TCGA-93-7347 | Higher-risk |
| TCGA-69-8253 | Higher-risk |
| TCGA-78-7158 | Higher-risk |
| TCGA-93-8067 | Higher-risk |
| TCGA-55-A4DF | Higher-risk |
| TCGA-55-8090 | Higher-risk |
| TCGA-97-7554 | Higher-risk |
| TCGA-44-6775 | Higher-risk |
| TCGA-62-8395 | Higher-risk |
| TCGA-44-6145 | Higher-risk |
| TCGA-44-3919 | Higher-risk |
| TCGA-55-7725 | Higher-risk |
| TCGA-55-6642 | Higher-risk |
| TCGA-62-A46O | Higher-risk |
| TCGA-05-4432 | Higher-risk |
| TCGA-L9-A443 | Higher-risk |
| TCGA-73-4668 | Higher-risk |
| TCGA-97-7552 | Higher-risk |
| TCGA-97-8174 | Higher-risk |
| TCGA-05-4398 | Higher-risk |
| TCGA-62-8402 | Higher-risk |
| TCGA-78-8648 | Higher-risk |
| TCGA-95-A4VP | Higher-risk |
| TCGA-97-8547 | Higher-risk |
| TCGA-49-AAQV | Higher-risk |
| TCGA-64-5774 | Higher-risk |
| TCGA-86-A4JF | Higher-risk |
| TCGA-83-5908 | Higher-risk |
| TCGA-93-A4JN | Higher-risk |
| TCGA-62-A470 | Higher-risk |
| TCGA-55-7994 | Higher-risk |
| TCGA-55-8615 | Higher-risk |
| TCGA-99-7458 | Higher-risk |
| TCGA-55-8299 | Higher-risk |
| TCGA-86-7711 | Higher-risk |
| TCGA-44-2662 | Higher-risk |
| TCGA-86-8279 | Higher-risk |
| TCGA-55-6987 | Higher-risk |
| TCGA-38-6178 | Higher-risk |
| TCGA-44-7670 | Higher-risk |
| TCGA-50-5066 | Higher-risk |
| TCGA-55-8619 | Higher-risk |
| TCGA-64-5781 | Higher-risk |
| TCGA-86-8278 | Higher-risk |
| TCGA-73-4675 | Higher-risk |
| TCGA-NJ-A55O | Higher-risk |
| TCGA-55-7727 | Higher-risk |
| TCGA-49-6761 | Higher-risk |
| TCGA-75-6212 | Higher-risk |
| TCGA-50-6590 | Higher-risk |
| TCGA-69-7763 | Higher-risk |
| TCGA-MP-A4TD | Higher-risk |
| TCGA-95-7043 | Higher-risk |
| TCGA-75-5125 | Higher-risk |
| TCGA-44-A47G | Higher-risk |
| TCGA-55-7283 | Higher-risk |
| TCGA-97-8175 | Higher-risk |
| TCGA-44-A479 | Higher-risk |
| TCGA-44-2655 | Higher-risk |
| TCGA-38-7271 | Higher-risk |
| TCGA-62-A472 | Higher-risk |
| TCGA-44-2659 | Higher-risk |
| TCGA-50-6592 | Higher-risk |
| TCGA-93-A4JQ | Higher-risk |
| TCGA-50-5946 | Higher-risk |
| TCGA-86-8671 | Higher-risk |
| TCGA-44-2656 | Higher-risk |
| TCGA-69-7973 | Higher-risk |
| TCGA-53-7626 | Higher-risk |
| TCGA-55-6968 | Higher-risk |
| TCGA-MP-A4TF | Higher-risk |
| TCGA-62-A46Y | Higher-risk |
| TCGA-55-7574 | Higher-risk |
| TCGA-78-7152 | Higher-risk |
| TCGA-4B-A93V | Higher-risk |
| TCGA-95-A4VN | Higher-risk |
| TCGA-55-A48Y | Higher-risk |
| TCGA-55-7724 | Higher-risk |
| TCGA-05-4417 | Higher-risk |
| TCGA-95-A4VK | Higher-risk |
| TCGA-44-8119 | Higher-risk |
| TCGA-50-6591 | Higher-risk |
| TCGA-55-6979 | Higher-risk |
| TCGA-49-AAR9 | Higher-risk |
| TCGA-L9-A5IP | Higher-risk |
| TCGA-50-7109 | Higher-risk |
| TCGA-55-8089 | Higher-risk |
| TCGA-64-1680 | Higher-risk |
| TCGA-95-7567 | Higher-risk |
| TCGA-38-4632 | Higher-risk |
| TCGA-55-7914 | Higher-risk |
| TCGA-MP-A4TK | Higher-risk |
| TCGA-64-1679 | Higher-risk |
| TCGA-05-5428 | Higher-risk |
| TCGA-55-6971 | Higher-risk |
| TCGA-38-4628 | Higher-risk |
| TCGA-78-7539 | Higher-risk |
| TCGA-MP-A4SY | Higher-risk |
| TCGA-78-7161 | Higher-risk |
| TCGA-55-7911 | Higher-risk |
| TCGA-49-4487 | Higher-risk |
| TCGA-55-6983 | Higher-risk |
| TCGA-44-2665 | Higher-risk |
| TCGA-86-8075 | Higher-risk |
| TCGA-NJ-A4YI | Higher-risk |
| TCGA-55-7816 | Higher-risk |
| TCGA-44-3398 | Higher-risk |
| TCGA-55-7284 | Higher-risk |
| TCGA-49-6743 | Higher-risk |
| TCGA-55-8085 | Higher-risk |
| TCGA-86-8674 | Higher-risk |
| TCGA-49-4510 | Higher-risk |
| TCGA-86-8672 | Higher-risk |
| TCGA-91-6830 | Higher-risk |
| TCGA-86-6562 | Higher-risk |
| TCGA-95-7562 | Higher-risk |
| TCGA-93-A4JP | Higher-risk |
| TCGA-49-4490 | Higher-risk |
| TCGA-73-A9RS | Higher-risk |
| TCGA-49-4505 | Higher-risk |
| TCGA-MP-A4TC | Higher-risk |
| TCGA-44-2666 | Higher-risk |
| TCGA-44-6777 | Higher-risk |
| TCGA-50-5942 | Higher-risk |
| TCGA-49-6744 | Higher-risk |
| TCGA-62-8394 | Higher-risk |
| TCGA-55-7726 | Higher-risk |
| TCGA-73-4659 | Higher-risk |
| TCGA-55-A48Z | Higher-risk |
| TCGA-53-A4EZ | Higher-risk |
| TCGA-MP-A4TE | Higher-risk |
| TCGA-50-8459 | Higher-risk |
| TCGA-69-7761 | Higher-risk |
| TCGA-05-4384 | Higher-risk |
| TCGA-71-8520 | Higher-risk |
| TCGA-50-5941 | Higher-risk |
| TCGA-05-4402 | Higher-risk |
| TCGA-05-4427 | Higher-risk |
| TCGA-91-7771 | Higher-risk |
| TCGA-55-7907 | Higher-risk |
| TCGA-97-A4M3 | Higher-risk |
| TCGA-44-3396 | Higher-risk |
| TCGA-69-7760 | Higher-risk |
| TCGA-64-1677 | Higher-risk |
| TCGA-44-6774 | Higher-risk |
| TCGA-69-7974 | Higher-risk |
| TCGA-J2-8194 | Higher-risk |
| TCGA-86-7713 | Higher-risk |
| TCGA-MP-A4T9 | Higher-risk |
| TCGA-J2-8192 | Higher-risk |
| TCGA-50-6595 | Higher-risk |
| TCGA-55-6982 | Higher-risk |
| TCGA-97-8176 | Higher-risk |
| TCGA-50-5051 | Higher-risk |
| TCGA-55-7227 | Higher-risk |
| TCGA-05-4390 | Higher-risk |
| TCGA-55-7910 | Higher-risk |
| TCGA-73-4666 | Higher-risk |
| TCGA-55-8505 | Higher-risk |
| TCGA-44-7661 | Higher-risk |
| TCGA-99-8033 | Higher-risk |
| TCGA-50-6593 | Higher-risk |
| TCGA-64-5815 | Higher-risk |
| TCGA-75-6214 | Higher-risk |
| TCGA-73-4670 | Higher-risk |
| TCGA-86-7701 | Higher-risk |
| TCGA-53-7624 | Higher-risk |
| TCGA-78-7220 | Higher-risk |
| TCGA-64-5779 | Higher-risk |
| TCGA-69-7978 | Higher-risk |
| TCGA-86-8055 | Higher-risk |
| TCGA-73-4676 | Higher-risk |
| TCGA-86-7714 | Higher-risk |
| TCGA-78-7145 | Higher-risk |
| TCGA-49-6742 | Higher-risk |
| TCGA-62-8398 | Higher-risk |
| TCGA-55-A490 | Higher-risk |
| TCGA-55-8205 | Higher-risk |
| TCGA-78-7536 | Higher-risk |
| TCGA-55-6712 | Higher-risk |
| TCGA-78-7148 | Higher-risk |
| TCGA-44-6779 | Higher-risk |
| TCGA-55-7815 | Higher-risk |
| TCGA-50-5939 | Higher-risk |
| TCGA-55-6981 | Higher-risk |
| TCGA-78-7166 | Higher-risk |
| TCGA-38-4629 | Higher-risk |
| TCGA-78-7150 | Higher-risk |
| TCGA-55-6970 | Higher-risk |
| TCGA-05-4382 | Higher-risk |
| TCGA-05-5425 | Higher-risk |
| TCGA-91-6848 | Higher-risk |
| TCGA-50-6673 | Higher-risk |
| TCGA-49-4506 | Higher-risk |
| TCGA-05-4424 | Higher-risk |
| TCGA-55-6975 | Higher-risk |
| TCGA-78-8660 | Higher-risk |
| TCGA-49-4507 | Higher-risk |
| TCGA-05-4415 | Higher-risk |
| TCGA-50-5072 | Higher-risk |
| TCGA-64-5775 | Higher-risk |

# Supplementary Figures


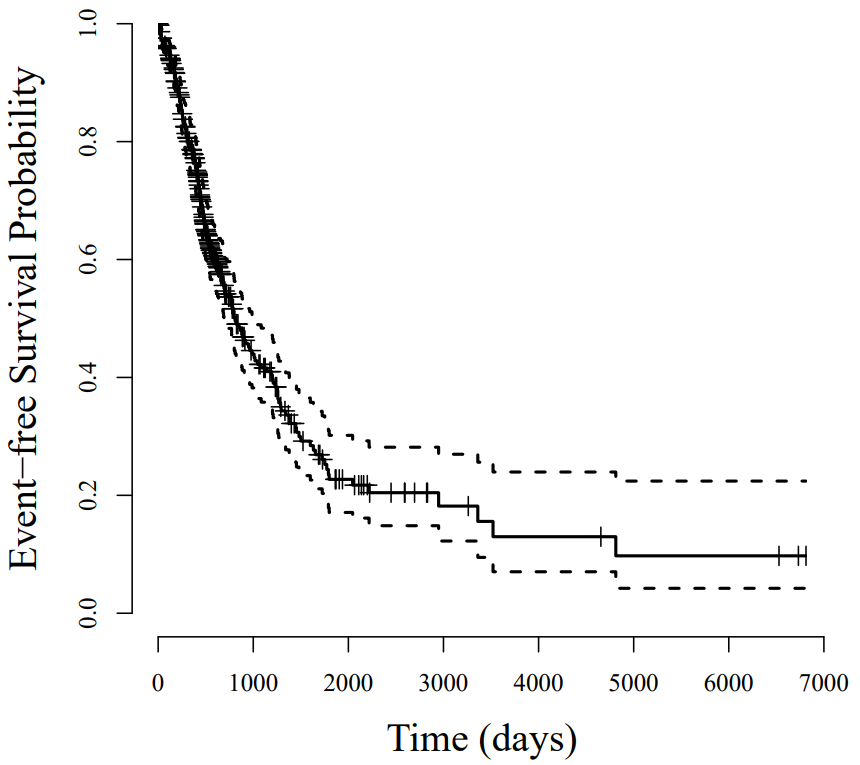


Figure S1: The Kaplan-Meier curve for EFS of 408 patients enrolled in this study.

Figure S2: The C-index of models developed based on single type of data (A) combined feature sets (B) and models with interaction covariates (C). The white box summarized C-index for models selected from cross-validation (1 standard error rule), while the gray box for those from testing. Abbreviations can be referred to Table S2.


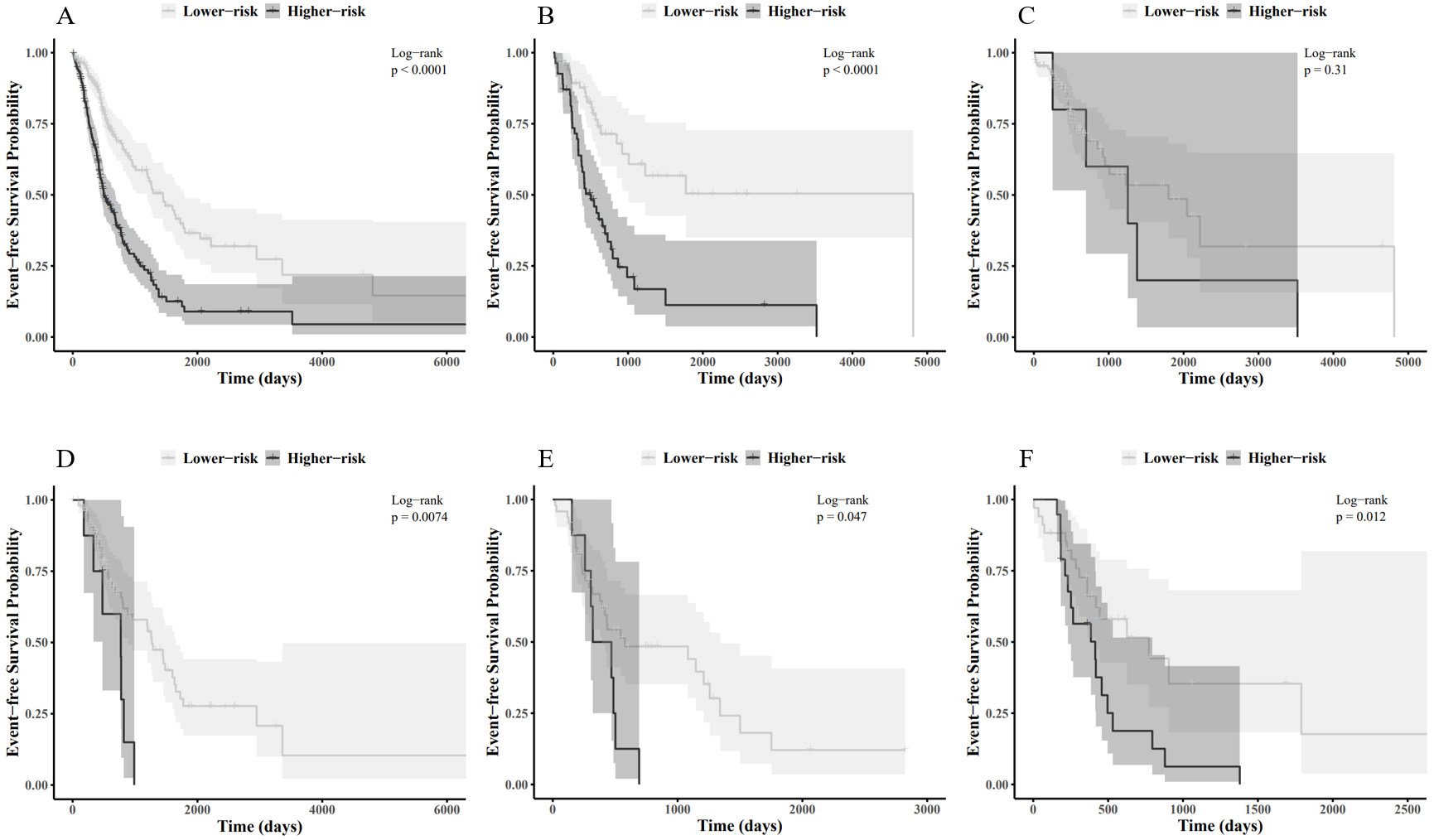


Figure S3: Kaplan-Meier curve of higher- and lower-risk subgroups stratified by cln-score within different sets of patients. The sets from A to F are: A, all patients; B, testing set; C, patients in AJCC pathologic tumor stage IA; D, patients in AJCC pathologic tumor stage IB; E, patients in AJCC pathologic tumor stage IIB; F, patients in AJCC pathologic tumor stage IIIA.

Figure S4: Barplot summary of inferred relative fractions of cell types (A) and volcano plot summary for the significance of difference in immune cellular compositions between the higher and lower-risk subgroup patients (B).
